# The Potential of Guanidino Acetic Acid to Reduce the Occurrence and Severity of Broiler Muscle Myopathies

**DOI:** 10.3389/fphys.2020.00909

**Published:** 2020-08-14

**Authors:** Edgar Orlando Oviedo-Rondón, Hernan Alejandro Córdova-Noboa

**Affiliations:** Prestage Department of Poultry Science, North Carolina State University, Raleigh, NC, United States

**Keywords:** guanidino acetic acid, myopathy, wooden breast, white striping, broiler chicken, meat yield, meat quality

## Abstract

Guanidinoacetic acid (GAA) is the biochemical precursor of creatine, which, in its phosphorylated form, is an essential high-energy carrier in the muscle. Although creatine has limited stability in feed processing, GAA is well established as a source of creatine in the animal feed industry. Published data demonstrate beneficial effects of GAA supplementation on muscle creatine, energy compounds, and antioxidant status, leading to improvements in broiler body weight gain, feed conversion ratio, and breast meat yield. Although increases in weight gain and meat yield are often associated with wooden breast (WB) and other myopathies, recent reports have suggested the potential of GAA supplementation to reduce the occurrence and severity of WB while improving breast meat yield. This disorder increases the hardness of the *Pectoralis major* muscle and has emerged as a current challenge to the broiler industry worldwide by impacting meat quality. Genetic selection, fast-growth rates, and environmental stressors have been identified to be the main factors related to this myopathy, but the actual cause of this disorder is still unknown. Creatine supplementation has been used as a nutritional prescription in the treatment of several muscular myopathies in humans and other animals. Because GAA is a common feed additive in poultry production, the potential of GAA supplementation to reduce broiler myopathies has been investigated in experimental and commercial scenarios. In addition, a few studies have evaluated the potential of creatine in plasma and blood enzymes related to creatine to be used as potential markers for WB. The evidence indicates that GAA could potentially minimize the incidence of WB. More data are warranted to understand the factors affecting the potential efficacy of GAA to reduce the occurrence and severity of myopathies.

## Introduction

Genetic selection in poultry has progressed continuously since the early 1960s, resulting in faster growth rates and higher meat production (Havenstein et al., 2003a, b). Over the past 60 years, body weight gain has increased fourfold from 1957 to 2005 with a simultaneous 50% reduction in feed conversion ratio and 79% higher *Pectoralis major* yield in males and 85% in females (Zuidhof et al., 2014). At the same time, the growth, development, structure, and overall metabolism of muscles have been modified by such selection, which has probably resulted in modifications affecting biochemical and sensory characteristics of meat (Petracci et al., 2015, 2019). Several studies have documented that fast-growing strains exhibit higher incidences of idiopathic or inflammatory myopathies and a greater susceptibility for stress-induced myopathies (Bailey et al., 2015; Chen et al., 2019; Montagna et al., 2019). These myopathies include muscle disorders, such as white striping (Kuttappan et al., 2013a, b), spaghetti muscle (Baldi et al., 2018; Montagna et al., 2019; Petracci et al., 2019), and wooden breast (WB; Owens et al., 2009; Petracci and Cavani, 2012; Bailey et al., 2015; Russo et al., 2015; Trocino et al., 2015), which significantly compromise chicken meat quality. WB is a pectoral myopathy in broilers that has been reported worldwide in the poultry industry. WB is macroscopically characterized by hardness of the *Pectoralis major* muscle often accompanied by pale color, greater drip, and cooking losses, and shear force in comparison to unaffected breast samples (Mudalal et al., 2015; Trocino et al., 2015; Sihvo et al., 2017; Sihvo, 2019). Affected filets are downgraded and have to be transformed into processed meat products, causing considerable economic losses (Mudalal et al., 2015; Petracci et al., 2015, 2019; Cruz et al., 2017). It has been proposed that some white striping lesions progress to become WB (Griffin et al., 2018).

The specific origin of these myopathies has not been well elucidated. Recent findings have linked their onset with hypoxia (Malila et al., 2019) due to reduced vascularization (Sihvo et al., 2018), phlebitis (Papah et al., 2017; Chen et al., 2019), and glucolipotoxicity (Mutryn et al., 2015; Abasht et al., 2016; Lake and Abasht, 2020). The final result is a muscle with defective energy-generating pathways combined with a deficiency and/or dysfunction of tissue ATPases, having consequences in myodegeneration and on muscle fiber contraction degree (Baldi et al., 2020). Genetics and factors related to early development, environment, and nutrition could be involved in the onset of these conditions (Bailey et al., 2015; Montagna et al., 2019; Petracci et al., 2019).

Currently, nutritional strategies to reduce the incidence of myopathies in high-yielding broiler chickens have shown sparse or minimal success. Generally, the reduction in the severity of myopathies has been associated with growth rate reduction (Petracci et al., 2015, 2019; Aviagen, 2019). Recent studies have reported that the dietary inclusion of guanidino acetic acid (GAA) partially ameliorated the occurrence and severity of WB myopathy while maintaining or improving live performance and breast meat yield (Córdova-Noboa et al., 2018a, b; Aviagen, 2019; Vargas, 2019). GAA is a metabolite precursor of creatine, a central energy molecule in muscles. However, only one experiment showed significant effects on white striping, and no effects were observed on spaghetti muscle. Several studies evaluating the use of creatine as a treatment for muscular dystrophies, and neurodegenerative diseases in humans have demonstrated positive outcomes (Hultman et al., 1996; Pearlman and Fielding, 2006; Kley et al., 2013). The hypothesized mechanisms by which GAA could be used to prevent broiler myopathies are revised throughout this review.

## Endogenous Production of GAA and Creatine Biosynthesis

**FIGURE 1:**
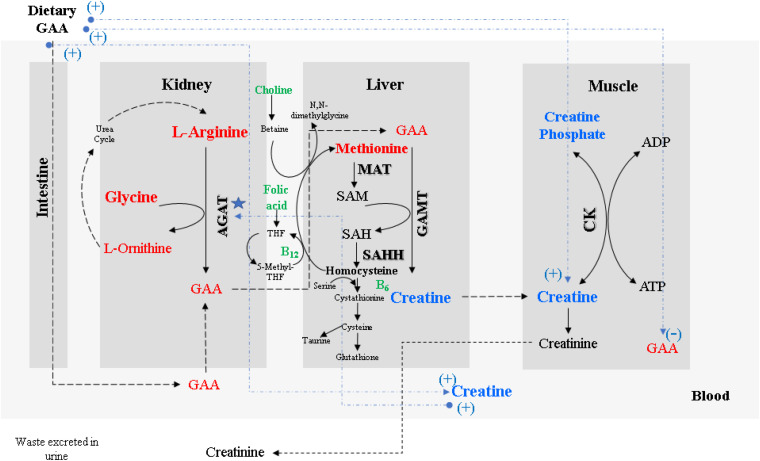
Metabolism of GAA and formation of muscular creatine. Dashed lines in blue color show the expected responses of GAA supplementation on broiler muscle and blood GAA and creatine concentrations. (Adapted from Wyss and Kaddurah-Daouk, 2000; Krueger et al., 2010; European Food Safety Authority [EFSA], 2016; Ostojic, 2016).

The chemical nomenclature for guanidino acetic acid (GAA) is N-[aminoiminomethyl]-glycine, also known as glycocyamine or guanidinoacetate. This compound was first isolated from the urine of dogs and humans and has been used as a therapeutic agent since the 1950s (Borsook and Borsook, 1951). Figure 1 describes the general metabolism of GAA with enzymes, amino acids, and vitamins involved. GAA is endogenously produced from arginine (Arg) and glycine (Gly) by a reaction catalyzed by the enzyme L-arginine:glycine amidinotransferase (AGAT; EC 2.1.4.1). Even though this reaction mainly occurs in the kidney and pancreas, some studies have reported that certain endogenous synthesis of GAA outside of the kidneys could be considerable (Ostojic, 2016). After being transported to the liver and pancreas, the enzyme guanidinoacetate methyltransferase (GAMT; EC 2.1.1.2) catalyzes the methylation reaction between GAA and S-adenosyl-methionine (SAM), yielding creatine and S-adenosyl-homocysteine also known as SAH (Wyss and Kaddurah-Daouk, 2000). SAM is generated from methionine (Met) by the enzyme methionine adenosyltransferase (MAT; EC 2.5.1.6). SAH is converted to adenosine and homocysteine by S-adenosyl-homocysteine hydrolase (SAHH; EC 3.3.1.1, also called adenosylhomocysteinase). Although SAH is recycled back to SAM via homocysteine and methionine in a reaction catalyzed by folic acid and vitamin B_12_, creatine is transported to the target cells, mainly the muscle, brain, and testes. In these tissues, it is phosphorylated by creatine kinase (CK; EC 2.7.3.2) to produce phosphocreatine. The latter is a high-energy phosphate store for skeletal muscles and the brain (Ostojic, 2015a) to immediately replenish adenosine triphosphate (ATP) from adenosine diphosphate (ADP) at times of ATP depletion.

The formation of GAA catalyzed by AGAT is considered the rate-limiting step for creatine biosynthesis because AGAT is subject to feedback inhibition by elevated blood creatine and ornithine concentrations (Wyss and Kaddurah-Daouk, 2000; De Groot et al., 2018). This inhibition is most likely in place to avoid unnecessary metabolic burden as well as to spare valuable amino acids used for creatine synthesis, namely Gly, Arg, and Met. However, Arg supplementation can increase muscle creatine in chickens, but additional Gly or Met does not have this effect (Khajali et al., 2020). There is evidence proposing that AGAT expression is upregulated when the body is deficient in folic acid, hence causing a shortage of SAM followed by low serum concentration of creatine (Wyss and Kaddurah-Daouk, 2000). Conversely, a different response is expected when the higher serum concentration of creatine is present due to either an endogenous source or by dietary uptake, which results in a reduction of the mRNA content and enzyme concentration, consequently reducing the enzymatic activity of AGAT. The evidence suggests that regulation of AGAT expression occurs at a pre-translational level (Van Pilsum et al., 1972; Stead et al., 2001; Derave et al., 2004). The creatine biosynthesis pathway is essential due to the role of phosphocreatine in cellular metabolism.

Phosphocreatine supplies energy for cellular activities and moderates the accumulation of ADP from ATP during high rates of cellular metabolism (Wallimann et al., 2011). Creatine and phosphocreatine degrade irreversibly to creatinine, which is excreted in the urine. The magnitude of this daily degradation has been estimated to roughly 1.7% of the total body pool of creatine and phosphocreatine (Wyss and Kaddurah-Daouk, 2000; Post et al., 2019). However, Tossenberger et al. (2016) suggest a lower daily degradation in modern broilers based on balance studies. Therefore, creatine must be refilled regularly from the diet or produced *de novo* from GAA, particularly in younger animals in which the requirement is higher due to the expanding muscle mass (Wyss and Kaddurah-Daouk, 2000; Tossenberger et al., 2016). Khajali et al. (2020) proposes that a 21-day-old chicken of 985 g and average body weight with a daily weight gain of 75 g would require 169 mg of creatine. Muscle creatine content is a good indicator of the incorporation of dietary creatine. Generally, more than 95% of the creatine pool is present in the muscles, and the remaining portion is located in the heart and brain. Broiler muscles contain 4.5 g/kg of creatine (Tossenberger et al., 2016; Majdeddin et al., 2018). During pathological situations, the creatine balance might be disturbed by metabolic disorders or renal failure (Post et al., 2019).

### Role of GAA and Creatine in Muscle Development and Activity

Guanidinoacetic acid has been related to muscle function mainly by its role in the formation of creatine. Nevertheless, an *in vitro* study conducted by Wang et al. (2018) using C2C12 cells, which are an immortalized mouse myoblast cell line, indicated that GAA stimulated myogenic differentiation 1 (MyoD) and myogenin (MyoG) mRNA expression increasing the myotube fusion rate. Myoblast fusion is a mechanism of increasing muscle mass without increasing muscle myofiber number. Additionally, GAA supplementation promoted myotube growth through an increase in total myosin heavy chain (MyHC) protein level, myotube thickness, and gastrocnemius muscle cross-sectional area. Finally, GAA promoted myoblast differentiation through MicroRNA (miR) -133a-3p and miR-1a-3p-induced activation of the AKT/mTOR/S6K signaling pathway. These miR are post-transcriptional regulators that play a crucial role in nutrient-mediated myogenesis (Wang et al., 2018). In another *in vitro* study conducted by Deldicque et al. (2005), supplementation with creatine to the same C2C12 cells increased insulin growth factor, IGF-I, and IGF-II mRNA expression by 30 and 40%, respectively. It has been shown that IGFs activate the PI3K-Akt-mTOR, p38 MAPK, and Erk1/2 MAPK pathways (Jiao et al., 2013).

Skeletal muscle contraction has a high-energy demand, and ATP is the immediate energy source. During this process, ATP is hydrolyzed to ADP and must be continuously replenished. With the fast increase in energy demand, the high-energy storage compound phosphocreatine is broken down to provide phosphate to ADP restoring ATP (Wyss and Kaddurah-Daouk, 2000). The creatine/phosphocreatine equilibrium is catalyzed by the enzyme CK. Phosphocreatine is accumulated in the muscles at times of rest and provision of phosphate to refuel ATP from ADP at times of energy demand (Balsom et al., 1994; Nabuurs et al., 2013). Creatine is transported freely in the blood and is mainly absorbed into muscle, brain, and testes (Guimbal and Kilimann, 1993; Wyss and Kaddurah-Daouk, 2000).

In addition to acting as a temporary energy buffer, the creatine/phosphocreatine system also serves other functions in skeletal muscle metabolism. For example, creatine may play a part in regulating muscle protein metabolism (Parise et al., 2001; Nabuurs et al., 2013). Phosphocreatine acts as a carrier, transporting high energy ATP from the mitochondria (production site) to various ATPase sites in the cytosol, especially in tissues of high-energy needs, such as brain and muscles (Balsom et al., 1994; Wyss and Kaddurah-Daouk, 2000). This process is also called the phosphocreatine “shuttle” system (Guimarães-Ferreira, 2014).

### GAA and Creatine in Muscle Disorders

In the early 1900s, it was observed that human patients with muscle diseases retained less creatine than healthy individuals (Hunter, 1928). In 1977, Fitch found that patients with muscle disease had lower cytosolic levels of creatine and phosphocreatine and suggested that this condition might be caused by a failure in the mechanism that retains creatine in the muscle (Fitch, 1977; Nabuurs et al., 2013). Another study also found that patients with neuromuscular disorders can have lower phosphocreatine and creatine levels in skeletal muscle than healthy subjects (Tarnopolsky et al., 2001). Consequently, dietary supplementation of creatine has been used as a pharmacological treatment for a variety of neuromuscular diseases (Tarnopolsky, 2007; Nabuurs et al., 2013). In human trials, creatine in muscles increased with creatine supplementation, and the magnitude of the increase appeared to be greater in patients with low endogenous stores of creatine (Harris et al., 1992; Tarnopolsky, 2007; Nabuurs et al., 2013). Reports indicate that short- and/or medium-term creatine supplementation recovers functional performance and strength in muscular dystrophy and idiopathic inflammatory myopathy (Tarnopolsky, 2007; Kley et al., 2013).

Broilers with WB have a dramatic reduction in ATP concentrations in the early postmortem period. This finding indicated a faulty ATP-generating pathway that might be related to the higher ultimate pH in WB samples (Baldi et al., 2020). In muscles with low ATP and creatine, GAA can be a direct substrate and thoroughly saturate CK and serve as compensatory phosphagen. Under these circumstances, phosphorylated GAA may play a role as a phosphocreatine mimetic and a substitute energy donor (Ostojic, 2015b, 2016). This mechanism could be the first mode of action of GAA to mitigate the development of myopathies. However, when the availability of creatine is unhindered, the potential of GAA to act as a substrate for CK is likely negligible. At normal physiological levels, GAA competes with creatine, but it is known that the flux *in vitro* through the CK reaction is ∼100 times lower for GAA as compared to creatine. The low affinity of CK for GAA is probably due to the lack of the N-methyl group, which is considered an essential structural feature for the CK reaction (Ostojic, 2015a).

Recently, Ostojic (2015a, 2016) summarized some other physiological roles of GAA in humans that might be relevant as well in animals to reduce the incidence of current poultry myopathies. GAA might stimulate insulin secretion and insulin sensitivity; spare dietary Arg and facilitate its use in protein synthesis and nitric oxide production; stimulate insulin, IGF-I, and glucagon release and vasodilation; modulate gamma-aminobutyric acid utilization and function; and affect oxidant-antioxidant balance. These effects are significant to mitigate myopathies observed in poultry. Wooden breast and other related broiler myopathies have been linked to metabolic disorders mainly due to glucolipotoxicity (Mutryn et al., 2015; Abasht et al., 2016; Lake and Abasht, 2020) and hypoxia (Malila et al., 2019). Insulin resistance causes low-grade inflammation, oxidative stress, and reduces vascularization in muscles (D’Souza et al., 2013), and consequently, increasing insulin sensitivity with GAA could be positive to mitigate myopathies. Higher dietary Arg levels (Arg:Lys ratio: + 30% in respect to the current recommendations) may play a role in reducing breast muscle abnormalities in broilers by increasing nitric oxide production, vascularization, and oxygenation and even helping with creatine metabolism (Zampiga et al., 2019). In contrast, Zampiga et al. (2018) observed that slight Arg increases were not adequate to mitigate myopathies. However, in practical terms of feed formulation, higher Arg:Lys ratios mean either higher dietary protein content or considerable supplementation of crystalline Arg. These factors impose environmental and economic limitations on implementing higher Arg levels in commercial poultry production.

Nevertheless, nitric oxide is a potent vasodilator compound that causes enhanced blood flow and oxygen supply to the muscle, as well as the removal of harmful catabolites (Khajali and Wideman, 2010), thereby possibly providing positive outcomes on myopathy occurrence (Zampiga et al., 2019). Finally, Amiri et al. (2019) and Nasiroleslami et al. (2018) concluded that GAA could improve the antioxidant status of broilers subject to heat or cold stress, respectively. In these studies, GAA supplementation improved the activity of antioxidant enzymes, such as superoxide dismutase and glutathione peroxidase, measured in serum and liver and decreased plasma levels of malondialdehyde. The antioxidant response should be preserved under stress conditions to minimize susceptibility to myopathy development (Lake and Abasht, 2020).

## GAA Used as Feed Additive for Poultry

In poultry nutrition, feed additives that improve energy utilization and naturally enhance muscle development are well accepted. Creatine could be one of these compounds. Creatine is found mostly in fresh meat, fish, and other animal products but is scarce in processed animal proteins (Balsom et al., 1994; Harris et al., 1997; Krueger et al., 2010; Dobenecker and Braun, 2015; De Groot et al., 2019; Khajali et al., 2020) and absent in plants (Khan and Cowen, 1977; Gábor et al., 1984; Krueger et al., 2010; Table 1). As a result, all-vegetable diets entirely lack creatine, and feeds containing animal proteins contain only a little of this semiessential nutrient (Michiels et al., 2012; De Groot et al., 2019; Khajali et al., 2020). The need for high-energy compounds such as phosphocreatine to support the fast growth rate of current broiler breeds is likely compromised as animal proteins are becoming less common in poultry diets.

**TABLE 1:** Concentration of guanidino acetic acid (GAA), creatine, and creatinine in mg/kg dry matter in different sources.

Ingredient	GAA	Creatine	Creatinine	References
		–		
Raw chicken breast		4,231		3
Boiled (60 min) chicken breast		2,973		3
Raw stewing beef		3,642		3
Boiled (60 min) stewing beef		2,856		3
Raw ox heart		2,948		3
Boiled (60 min) ox heart		2,017		3
Raw ox liver		309		3
Boiled (60 min) ox liver		202		3
Cod		3,000		4
Herring		6,500–10,000		4
Beef		4,500		4
Meat meal (*n* = 20)		885		8
Meat and bone meal	2.4 ± 1	89 (54–447), 885	868.8 ± 565	5
Meat and bone meal (*n* = 6)		199		8
Blood meal (*n* = 8)		67		8
Poultry by-products	4.0 ± 1	201.2 ± 108	1,467.1 ± 1,014	5
Poultry by-products (*n* = 2)		156		
Fish meal	2.0 ± 1	1,110.5 ± 808	2,406.5 ± 2,330	5
Fish meal		1,146		8
Unprocessed bone and raw food diet^1^ (*n* = 90)		1,318–8,548		6
Unprocessed prey^2^ (*n* = 15)		2,735–12,977		6
Corn	ND	ND	ND	5, 7
Wheat	ND	ND	ND	5
Sorghum	ND	ND	ND	5
Canola meal	ND	ND	ND	5

*^1^Raw single feedstuffs (cattle meat, head, larynx, lungs, lamb belly, and carcasses). ^2^Whole commercially bred or free-catch prey animals (mice, rats, birds, day-old chicken, fish, rabbits). ^3^Harris et al. (1997). ^4^Balsom et al. (1994). ^5^Krueger et al. (2010). ^6^Dobenecker and Braun (2015). ^7^Córdova-Noboa et al. (2018b). ^8^De Groot et al. (2019).*

Creatine is not an ideal feed additive due to its instability and cost (Baker, 2009). Consequently, GAA became attractive to the feed industry as a precursor of creatine due to its industrial properties as a compound with high stability in aqueous solutions (Vranes et al., 2017), and feed processing stability during pelleting and extruding (Van der Poel et al., 2018). Currently, GAA is an approved source of creatine in Europe (European Food Safety Authority [EFSA], 2009, 2016; EC(VO) 1768, 2016) as well as in the United States (FDA 21 CFR § 573.496).

The supplementation of GAA at 600 or 1200 mg/kg to broiler diets has promoted growth, enhanced breast meat yield and other carcass traits, and improved feed conversion ratio (Michiels et al., 2012; Mousavi et al., 2013; Heger et al., 2014; Tossenberger et al., 2016; De Groot et al., 2018; Khajali et al., 2020). The most consistent effect of GAA supplementation has been observed in feed conversion ratio with 4.5- and 8.8-point improvement in broilers supplemented with 600 and 1200 mg/kg, respectively (Khajali et al., 2020).

Dietary GAA has obtained interest to promote higher muscle creatine levels (Majdeddin et al., 2018; Ibrahim et al., 2019), which, in turn, have been associated with improved energetics (Ostojic, 2017; Sadra et al., 2018) and significant increases of high phosphate energy metabolites in broilers supplemented with GAA (Michiels et al., 2012; De Groot et al., 2018; Majdeddin et al., 2018). Improvements in energy utilization have been related to better feed utilization (Khajali et al., 2020). Heger et al. (2014) and Ale Saheb Fosoul et al. (2018) reported increases in energy utilization when GAA was supplemented to broiler diets with variable energy levels, which resulted in a better feed conversion ratio. Similar enhancements in feed and energy efficiency due to GAA supplementation have been observed by Mousavi et al. (2013) and Majdeddin et al. (2018).

**FIGURE 2:**
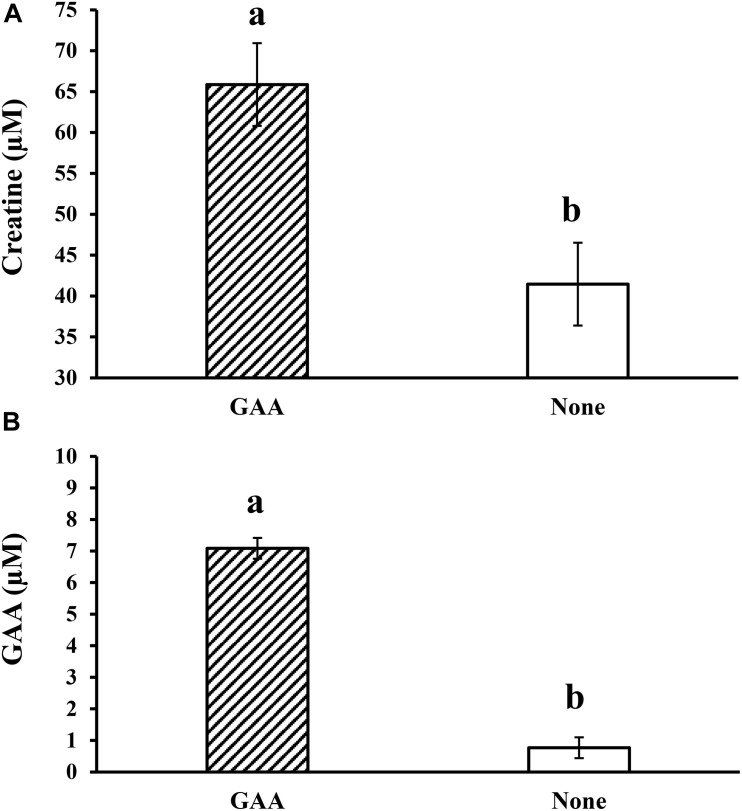
Effect of dietary inclusion of guanidino acetic acid (GAA) at 600 g/ton on **(A)** serum creatine concentration and **(B)** serum GAA concentration in 53-day old broiler chickens (adapted from Córdova-Noboa et al., 2018b).

The initial research (Beard and Barnes, 1931) conducted in young rats indicated that feeding 1 g of GAA for 4–6 h increased about 49% (5.94 ± 0.8 mg of creatine per g of muscle) the creatine concentration in muscle tissue from the hind legs over the control value (4.0 ± 0.2 mg of creatine per g of muscle). Compared to other supplements and purified amino acids studied (Ala, Gly, Aspartic acid, glutamic acid, Phe, Tyr, Leu, choline hydrochloride, creatine, His, casein, Val, Cys), GAA yielded the highest increment in creatine above control levels. Even when equivalent concentrations of creatine were supplemented to other treatment groups, the response was better for GAA (Beard and Barnes, 1931). In broilers fed diets with variable energy content and supplemented with GAA, Tabatabaei Yazdi et al. (2017) observed increased concentrations of phosphocreatine, ATP/ADP, and phosphocreatine/ATP ratios in breast muscle. Likewise, Córdova-Noboa et al. (2018b) found that the dietary GAA supplementation (600 g/ton) increased the serum concentrations of creatine and GAA in broiler chickens by 1.6 and 9.2 times, respectively, compared to non-supplemented broilers (Figure 2). In the same way, Nasiroleslami et al. (2018) had reported higher CK activity in GAA-supplemented broilers than in the control group. In a more recent publication, Ibrahim et al. (2019) demonstrate that dietary supplementation of GAA plus 0.4% methionine (GAA + 0.4% Met) increased carcass and breast meat yield and improved plasma and muscle creatine more than dietary creatine itself in mulard ducks.

In addition to the effects in muscle energetics, GAA also has Arg-sparing impacts as reported by Baker (2009); Dilger et al. (2013), Michiels et al. (2012), Ahmadipour et al. (2018a; 2018b; 2018c), and De Groot et al. (2018, 2019). Poultry are uricotelic animals, lacking enzymes to synthesize enough Arg *de novo* and, therefore, need a sufficient supply of Arg for creatine and nitric oxide production. Arg is especially required under metabolic, environmental, and immunological stress conditions (Khajali and Wideman, 2010). GAA is a source of creatine and, consequently, can spare Arg and, hence, is an excellent candidate to reduce myopathy occurrence and severity. GAA can leave more Arg available to improve muscle regeneration, vascularization, and vasodilatation for better oxygenation necessary to minimize WB (Sihvo et al., 2018; Sihvo, 2019). The Arg-sparing effect has been proven to be an efficient strategy to “supply” Arg in low protein diets and under heat stress (Amiri et al., 2019). This Arg-sparing effect is also important when feeding diets with high inclusion levels of feed ingredients with low Arg content or digestibility, such as wheat, canola meal, distiller’s dried grains with solubles, and sorghum (Khajali et al., 2020).

## The Potential of GAA to Reduce Myopathies in Poultry

Only a few studies have explored the feasibility of alleviating myopathies by supplementing broiler diets with GAA (Córdova-Noboa et al., 2018a, b; Aviagen, 2019; Vargas, 2019). In two separate experiments with male broilers, Córdova-Noboa et al. (2018a, b) demonstrated that supplementing with GAA (600 g/ton) improved breast meat yield and ameliorated WB myopathy in male Ross 708 broilers. However, no significant effects on white striping and spaghetti muscle were detected in both experiments. The positive results on WB incidence were mainly due to increasing the number of breast filets with low WB scores and decreasing the filets with more severe WB. The WB severity was evaluated by using the four-level scale (Table 2) developed by Tijare et al. (2016).

**TABLE 2:** Wooden breast (WB) scoring system and characteristics of broiler breast filets detected by palpation at 55 day of age (adapted from Tijare et al., 2016).

WB gross classification	Score	Characteristics
Normal	1	No hardness detected and flexible through
Low	2	Mild hardness specially in cranial region, flexible in caudal region
Moderate	3	Hardness throughout with some flexibility in mid to caudal region
Severe	4	Marked hardness, rigid throughout

**FIGURE 3:**
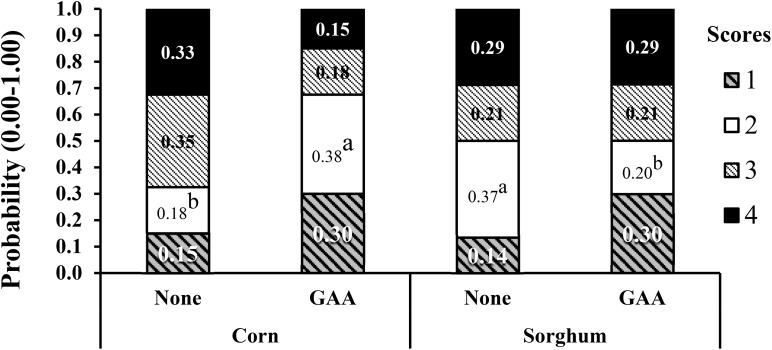
Effect of GAA supplementation (600 g/ton) in diets based on either corn or sorghum on the probability distribution for each wooden breast severity score in Ross-708 male broilers at 51 day of age. Means not sharing a common superscript (a-b) are significantly different (*P* < 0.05) by Tukey’s test. Each value represents the probability (0–1) of developing each severity score. Woody breast scores are based on a four-point scale (1 = normal, 2 = low, 3 = moderate, 4 = severe). Reproduced from Córdova-Noboa et al. (2018a) with the authors’ permission.

In the first experiment with corn or sorghum diets (Córdova-Noboa et al., 2018a), 40 breast samples per treatment, four per each experimental pen were evaluated. The addition of GAA roughly doubled the probability of normal breasts (score 0) from approximately 15 to 30% at 51 days of age (Figure 3) although the breast meat yield increased from 38.19 to 39.15% at 55 days of age only in broilers fed corn-based diets. No significant effect of GAA supplementation was detected on white striping and spaghetti muscle in both evaluations, and no significant impact of GAA was detected at 55 days of age in any of the myopathies evaluated. Heat stress during the last days of life affected the variability in the data, and only effects related to the type of grain were detected in that experiment.

**FIGURE 4:**
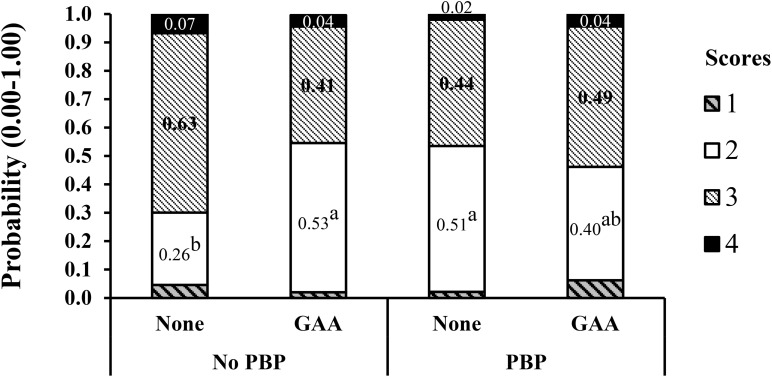
Interaction effect of dietary inclusion of poultry by-products (PBP) and GAA supplementation (600 g/ton) on the probability distribution for each wooden breast severity score in Ross-708 male broilers at 56 day of age. Means not sharing a common superscript (a-b) are significantly different (*P* < 0.05) by Tukey’s test. Each value represents the probability (0.0–1.0) of developing each severity score. Scores are based on a four-point scale (1 = normal, 2 = low, 3 = moderate, 4 = severe). Reproduced from Córdova-Noboa et al. (2018b) with authors’ permission.

In a similar way, in a second experiment (Córdova-Noboa et al., 2018b) in corn–soybean meal diets with or without poultry by-products (PBP), 64 breast meat samples per treatment, four from each experimental pen were scored. The improvement in WB score at 56 days of age with GAA supplementation was more evident in male broilers fed diets without PBP and probably lower in some digestible amino acids (Figure 4). In this case, the number of breast filets with low WB (score 2) doubled, and that of score 3 halved in the GAA-supplemented treatment only for broilers fed diets containing no PBP, reducing the more severe scores of 3 and 4. The breast meat yield increased significantly in the GAA-supplemented diets with PBP compared to the non-supplemented (32.35 vs. 31.74%).

In both experiments reported by Córdova-Noboa et al. (2018a, b), results were slightly different depending on dietary composition. Carvalho et al. (2013) reported that in corn–soybean meal diets with or without meat and bone meal, GAA supplementation only improved body weight gain when diets also contained 5% blood meal. Most likely, amino acid levels played a more prominent role than creatine in these diets because blood meal has even lower creatine content than meat and bone meal. Boney et al. (2019) observed that the inclusion of GAA in non-animal-protein diets did not affect breast meat yield; however, breast meat yield was reduced by 1.78 percentage points when broilers were provided the diet containing PBP meal devoid of GAA. Therefore, the variable effects in these experiments could be more related to specific nutrient digestibility than to feed ingredients or origin of the protein *per se*.

The degree of responses to GAA supplementation in live performance, breast meat yield, and muscle myopathy have varied by dietary levels of energy (Abudabos et al., 2014; Sadra et al., 2018) and nutrients such as Arg, Met, folic acid, vitamin E (Wyss and Kaddurah-Daouk, 2000; Dilger et al., 2013; De Groot et al., 2019; Ibrahim et al., 2019; Majdeddin et al., 2019; Khajali et al., 2020) and minerals (Vargas, 2019). The GAA or creatine action always depends on the dietary level of other nutrients to influence muscle metabolism due to its regulatory pathways (Figure 1). However, as was expected, the PBP-diets did not contain detectable levels of creatine due to the low creatine level in the PBP itself. Strikingly, both experiments indicated that GAA increased breast meat yield significantly in large broilers without augmenting the incidence of WB or other myopathies.

**FIGURE 5:**
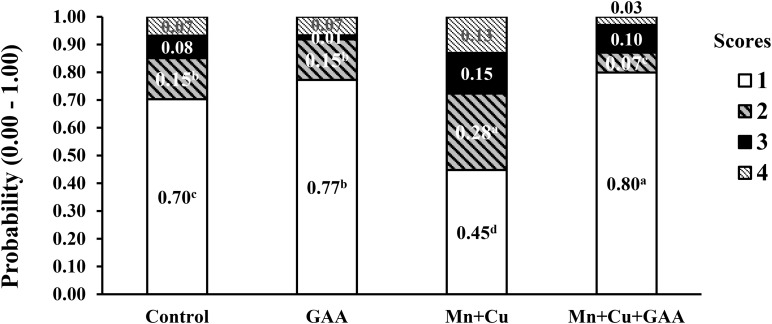
Effect of guanidinoacetic acid (GAA) and organic trace minerals (manganese, Mn, and copper, Cu) on the probability distribution for each wooden breast severity score in Ross-308 broilers at 42 day of age (adapted from Vargas, 2019).

**FIGURE 6:**
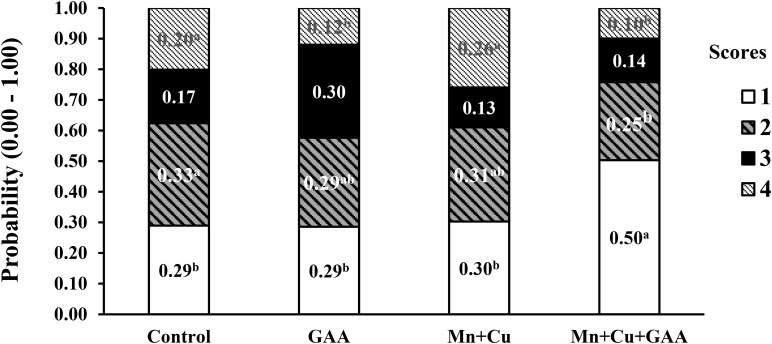
Effect of guanidino acetic acid (GAA) and organic trace minerals (manganese, Mn, and copper, Cu) on the probability distribution for each white striping severity score in Ross-308 broilers at 42 day of age (adapted from Vargas, 2019).

In a more recent experiment, Vargas (2019) evaluated the dietary supplementation GAA (600 g/ton) and chelated trace minerals (Mn and Cu) in Cobb 500 up to 42 days of age on performance and muscle myopathies (WB and white striping). Broilers were fed corn–soybean meal diets with PBP, soybean oil, and meat and bone meal (1.8 to 2.4%) in all diets and feather meal (1.50%) in the last feeding phase. A total of 120 carcasses per each treatment were evaluated for carcass and cut-up part yields and WB and white striping incidence, among other carcass quality parameters, in a commercial processing plant using a similar scoring system as described by Tijare et al. (2016). The presence of spaghetti muscle was not evaluated. Results indicated that GAA supplementation did not significantly affect live performance, carcass or meat yield, or physicochemical parameters of breast meat compared to the control treatment. The dietary inclusion of GAA + Mn + Cu increased the number of normal filets from 70.8% in the control treatment to 80.4% while reducing severe WB from 6.79 to 2.94% (Figure 5). More evident benefits on reducing WB were observed with GAA than with alternative supplements such as nucleotides and organic Mn and Cu minerals alone. The supplementation of GAA had the most significant reduction in WB score 2 compared to control (1.44 vs. 8.13%). White striping was also reduced by the combined inclusion of GAA + Mn + Cu (Figure 6). Normal filets in this treatment were 50.83% compared to 29.46% in the control treatment. Filets scored as severe white striping were 20.13% in the control treatment; the supplementation with GAA reduced them to 11.97%, and the addition of trace minerals GAA + Mn + Cu reduced them further to 9.98%. The data reported by Vargas (2019) indicated once again the relationship between GAA supplementation and the presence of other nutrients, in this case, trace minerals. In a larger sample size than Córdova-Noboa et al. (2018a, b), results from Vargas (2019) evidenced moderate positive effects of WB and white striping.

The early results reported by Córdova-Noboa et al. (2018a, b) generated interest in replicating these experiments on a commercial scale. A recent report by Aviagen (2019) described the use of GAA (600 g/ton) in three commercial-scale trials. The research, conducted in the United States demonstrated slight improvements (decrease) in WB severity at 49 days of age, but not at 56 days of age. The other two tests were conducted in Europe and showed a 17 and 31% decrease in the incidence of WB. These studies also indicate that growth performance and breast meat yield were increased in chickens fed GAA-diets while reducing WB incidence. However, there was no indication that white striping was affected in these commercial trials, and spaghetti muscle is not discussed in this report. In summary, GAA, as a feed additive, enhanced productivity of broilers and meat yield without exacerbating breast muscle myopathies.

These beneficial results of GAA on WB incidence and severity could be explained by its role on reducing insulin resistance, sparing Arg to increase vasodilation, improving antioxidant capacity (Ostojic, 2015a), and the energetic balance in muscles due to an increase in muscle creatine and as such help supply ATP and proper muscle function during growth (Baldi et al., 2020). Ibrahim et al. (2019) observed that GAA + 0.4% Met was superior to creatine to enhance gene expression of insulin growth factor-1, growth hormone, and muscle myogenin and downregulate myostatin in mulard ducks. These two hormones and two muscle transcription factors are affected in myopathies (Vignale et al., 2017; Brothers et al., 2019). Therefore, the positive effects of GAA on its regulation could aid in mitigating moderate WB. Ibrahim et al. (2019) also indicated that a possible shortage of methyl groups could be induced by long-term GAA supplementation in ducks, which could impair protein synthesis unless more methionine is added to the feed. In diets based on sorghum, the lower Arg, and Met digestibility as a consequence of kafirin and tannin presence could reduce the efficacy of GAA (Córdova-Noboa et al., 2018a). Therefore, attention to dietary factors seems essential, and more research is warranted to validate positive results with GAA.

## Creatine as a Marker for Wooden Breast Myopathy?

Finally, it is relevant to highlight that creatine and CK have been selected as markers of numerous myopathies in several species. The CK, alanine transaminase (ALT), and lactate dehydrogenase (LDH) concentrations in blood have been widely studied as blood markers to screen individuals for suspected muscle damage and necrosis in dogs (Tvarijonaviciute et al., 2017), horses (Söderqvist et al., 2013), and humans (Hathout et al., 2016). Therefore, it is already well established that blood concentrations of these enzymes are suitable biomarkers for myopathies.

In the case of broiler chickens, Kawasaki et al. (2018) found roughly three to four times greater, concentrations of plasma CK and L-aspartate aminotransferase (AST) in 20-day old chickens with severe WB than in chickens with normal breast filets (Table 3). Plasma CK and AST might also be helpful in detecting WB in young chickens, but the concentration of these enzymes is also increased in other broiler myopathies. Amaral et al. (2017) concluded that serum CK and AST concentrations were the only blood parameters that differed between broilers with dorsal cranial myopathy and healthy broilers. In that, myopathy of the *anterior latissimus dorsi* muscle is affected (Table 3).

**TABLE 3:** Plasma creatine kinase (CK) and L-aspartate aminotransferase (AST) concentrations in broilers with dorsal cranial myopathy or woody breast.

Parameter	Affected	Unaffected
	
	————–IU/L————–	
**Dorsal cranial myopathy^1^**		
CK*	54,091^a^	35,203^b^
AST**	409^a^	320^b^
**Woody breast^2^**		
CK**	42,360^a^	10,164^b^
AST**	356^a^	131^b^

^1^Adapted from Amaral et al. (2017). n = 28 broilers processed at 42 days of age. ^2^Adapted from Kawasaki et al. (2018). n = 10 broilers sacrificed at 20 and 29 days of age. * P < 0.05; ** P < 0.01; ^a,b^Mean values in the same line followed by different letter superscripts are significantly different (P < 0.05).

A study conducted by Córdova-Noboa (2015) compared the serum concentrations for different metabolites in 78 Ross 708 male broilers processed at 55 days of age. The results presented in Table 4 show that broilers affected with severe WB yielded higher blood concentrations of ALT, AST, LDH, and CK in comparison with samples from broilers that had normal, low, and moderate WB scores. The levels of LDH and CK almost doubled in broilers with severe (score 4) WB compared to broilers with no WB. In addition, the pairwise correlations indicate a moderate (*r* = 0.43–0.63) positive correlation (*P* < 0.001) of enzyme concentrations in plasma with WB scores (Table 4). Therefore, the levels of enzymes such as CK, LDH, AST, and ALT are potential markers of WB, and the accuracy for combined use in the detection of myopathies should be further evaluated. These biomarkers indicate once again the importance of muscle creatine and CK in WB myopathy and the relevance of GAA as potential feed additive to reduce its occurrence.

**TABLE 4:** Serum ALT, AST, LDH, and CK concentrations of broilers affected by different WB severity.

Enzymes (U/L)	Wooden breast score^1^				SEM	Pairwise correlation with WB
	1	2	3	4		r*
*N*	8	25	32	13		
ALT*	5.50^b^	5.42^b^	6.58^b^	8.89^a^	0.50	0.54
AST*	451.67^b^	511.05^b^	653.79^b^	888.25^a^	54.78	0.63
LDH*	1,803^b^	1,714^b^	2,175^b^	4,670^a^	470	0.46
CK*	26,740^c^	45,490^c^	67,826^b^	109,520^a^	10,459	0.53

* P < 0.001; ^a–c^Mean values in the same line followed by different letter superscripts are significantly different (P < 0.001). Adapted from Córdova-Noboa (2015).

## Conclusion

There is some evidence in the literature from three studies conducted at the experimental level (Córdova-Noboa et al., 2018a, b; Vargas, 2019) and three commercial scenarios (Aviagen, 2019), indicating that dietary supplementation of guanidino acetic acid (GAA) at 600 g/ton reduces the occurrence and ameliorates the severity of wooden breast in broilers. However, only one experiment indicated that GAA can significantly reduce the incidence or severity of white striping. There is no evidence that spaghetti muscle incidence or severity is affected by GAA supplementation, but these have not been evaluated extensively. The effects of higher levels of GAA supplementation (1200 g/ton) that have been proven to improve live performance and some muscle characteristics have not been evaluated in the incidence and severity of myopathies. Several research reports have indicated that GAA could support muscle development, function, and regeneration by improving energy homeostasis, increasing physiological levels of creatine in muscles in broilers and in many other species. Creatine in its phosphorylated form phosphocreatine regenerates ATP, the key molecule in muscle energy balance, contraction, protein metabolism, and development. GAA’s support of creatine production is of particular importance because commercial poultry feed is deficient in creatine, and poultry genetics are selected for higher muscle development rates, which makes them susceptible to myopathies. The levels of creatine in the muscle and the CK together with LDH in blood are closely related to WB, indicating the importance of creatine metabolism in myopathies. Research results have also shown that GAA may also potentially prevent broiler muscle degeneration by several mechanisms that include improving glucose metabolism, releasing IGF’s, sparing Arg to enhance protein formation, nitric oxide production, improving vascularization, vasodilatation, and oxygenation. Finally, it has been confirmed that GAA supplementation may stimulate myotube growth, reduce oxidative muscle stress, and allow muscle fiber regeneration.

## Author Contributions

EO-R and HC-N worked on the research projects presented and in the literature research conducted.

## Conflict of Interest

The authors declare that the research was conducted in the absence of any commercial or financial relationships that could be construed as a potential conflict of interest.
